# Acute appendicitis secondary to Enterobius vermicularis infection in a middle-aged man: a case report

**DOI:** 10.1186/1752-1947-5-559

**Published:** 2011-11-30

**Authors:** Stavros Panidis, Daniel Paramythiotis, Dimitris Panagiotou, Georgios Batsis, Spyridon Salonikidis, Vassiliki Kaloutsi, Antonios Michalopoulos

**Affiliations:** 1 First Propedeutic Department of Surgery, AHEPA University Hospital, Aristotle University of Thessaloniki, St Kyriakidi 1 54636, Thessaloniki, Greece; 2 Department of Pathology, Medical School, Aristotle University of Thessaloniki, St Kyriakidi 1 54636, Thessaloniki, Greece

## Abstract

**Introduction:**

Acute appendicitis due to *Enterobius vermicularis *is very rare, affecting mostly children. Whether pinworms cause inflammation of the appendix or just appendiceal colic has been a matter of controversy.

**Case presentation:**

A Caucasian 52-year-old man was referred to our Emergency Department with acute abdominal pain in his right lower quadrant. The physical and laboratory examination revealed right iliac fossa tenderness and leukocytosis with neutrophilia. An open appendectomy was performed. The pathological examination showed the lumen containing pinworms. Two oral doses of mebendazole were administered postoperatively. The follow-up to date was without incident and he was free of symptoms one year after the operation.

**Conclusion:**

The finding of *E. vermicularis *in appendectomy pathological specimens is infrequent. Parasitic infections rarely cause acute appendicitis, especially in adults.

One should keep in mind that the clinical signs of intestinal parasite infection may mimic acute appendicitis, although rare. A careful evaluation of symptoms such as pruritus ani, or eosinophilia on laboratory examination, could prevent unnecessary appendectomies.

## Introduction

*Enterobius vermicularis*, commonly known as pinworm or threadworm, is responsible for a widespread parasitic infection estimated to affect up to 209 million people worldwide [1]. Around 4% to 28% of children worldwide are reported to be infected [2-6]. Pinworms measure approximately 10 mm in length and live with their heads embedded in the right hemicolon and adjacent bowel [7]. Infection via the fecal-oral route is the most common route of human transfer, while eggs may remain viable for two to three weeks on clothing and bedding, facilitating easy spread among family members and groups of children [1].

*E. vermicularis *infection is usually asymptomatic. The most common symptom is pruritus in the perianal region, but infestation may also present with ileocolitis, enterocutaneous fistula, urinary tract infection, mesenteric abscesses, salpingitis and appendicitis [8]. The presence of pinworms in the appendix has been shown to cause symptoms mimicking appendicitis or appendiceal 'colic' [9-11] but frequently without any histological evidence of acute inflammation [8,12-14]. The presence of *E. vermicularis *is associated with chronic inflammatory infiltrates and eosinophilia [15].

We present the case of a 52-year-old man with right iliac fossa pain, who underwent appendectomy and the pathology revealed *E. vermicularis*.

## Case presentation

A 52-year-old Caucasian man was referred to our Emergency Department with acute abdominal pain in his right lower quadrant, mild fever, anorexia and nausea.

**Figure 1:**
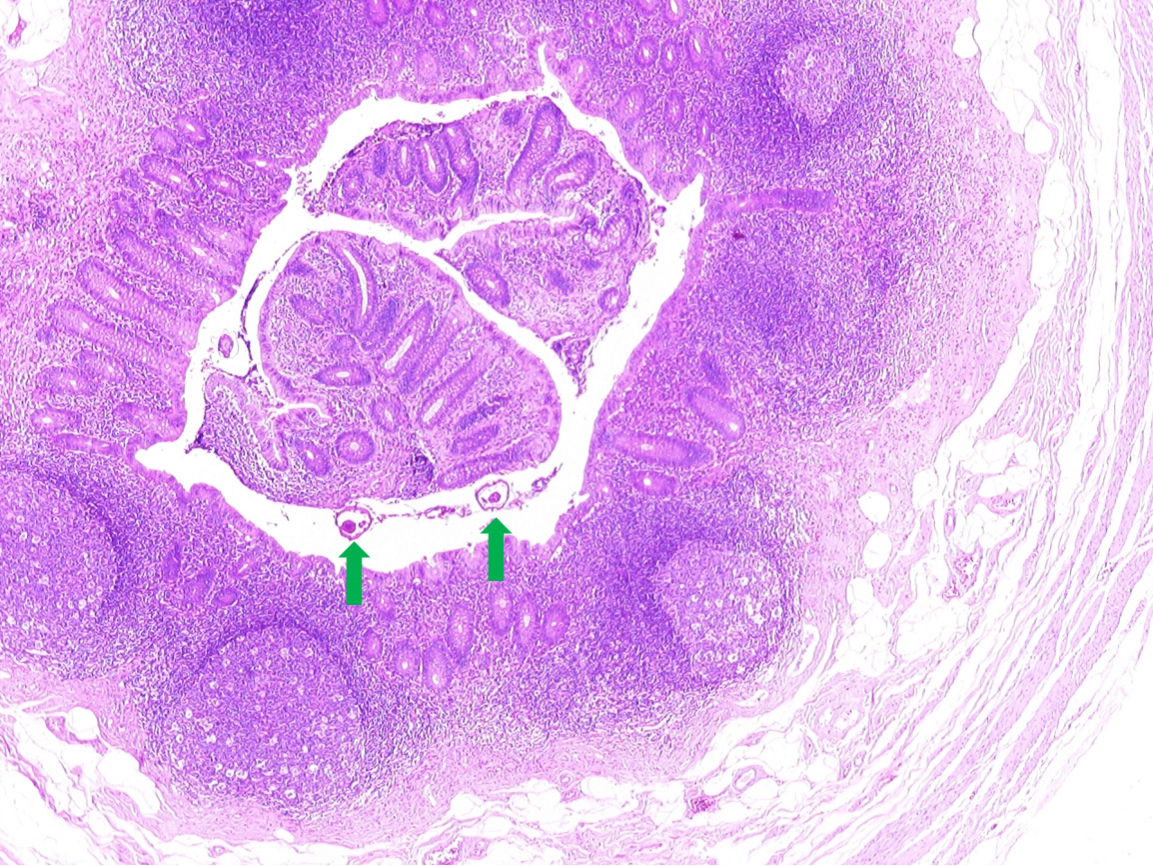
**Cross section through the appendix demonstrates the presence of three worms**. Hematoxylin and eosin stain, ×40 magnification.

**Figure 2:**
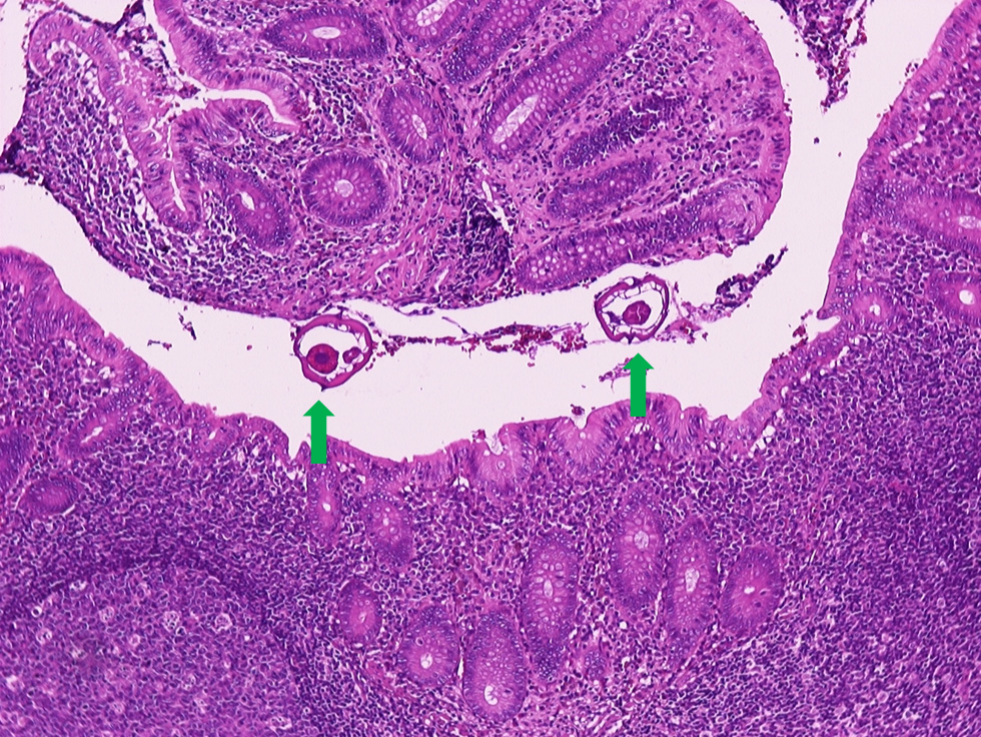
**Presence of worms in the appendiceal lumen**. The underlying mucosa essentially appears normal. Hematoxylin and eosin stain, ×100 magnification.

**Figure 3:**
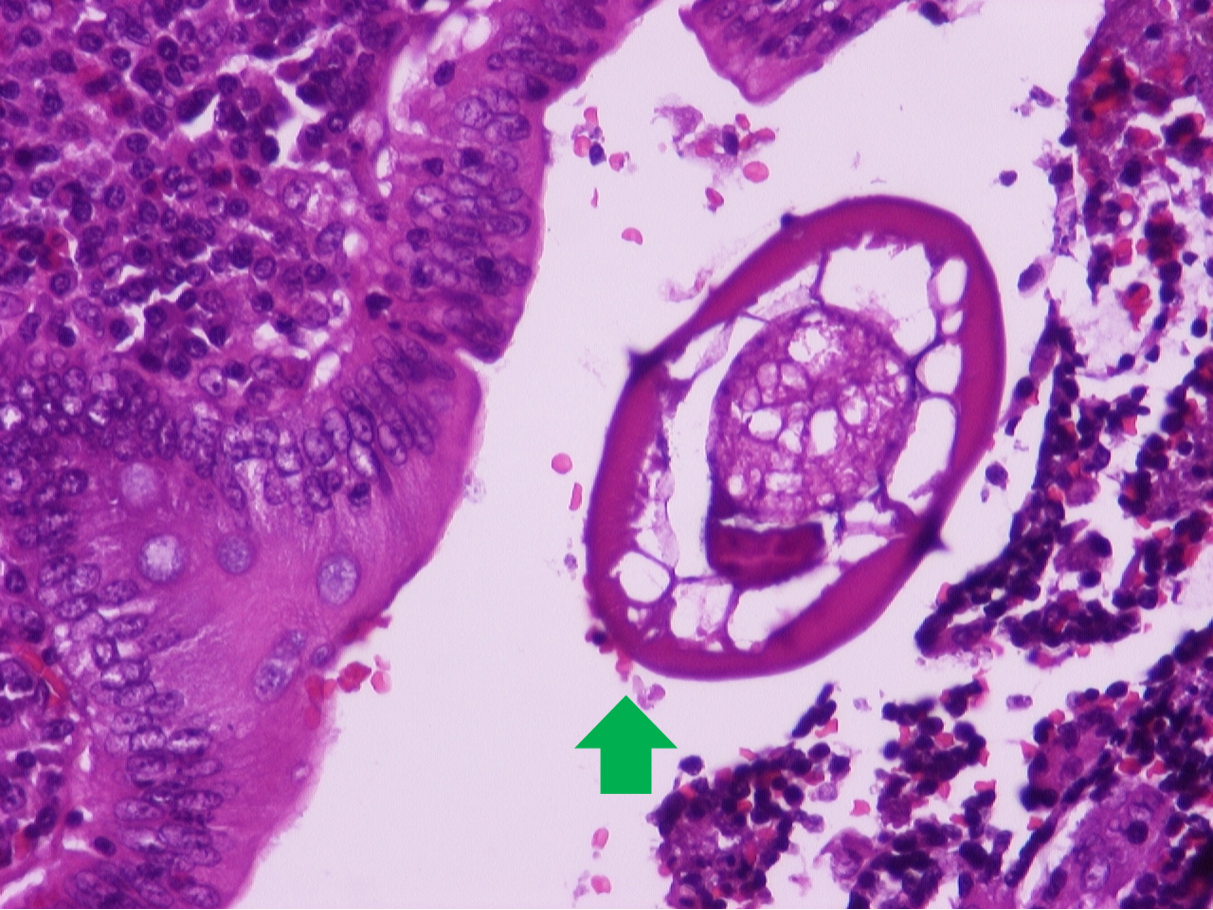
**Cross-section of *E. vermicularis *in the appendiceal lumen**. Note the characteristic pair of cuticular crests and the typical eggs. The mucosa shows a mild chronic inflammation with eosinophils. Hematoxylin and eosin stain, ×400 magnification.

A physical examination revealed right iliac fossa tenderness (McBurney's sign) and a positive Rovsing's sign. A laboratory examination showed an elevated white blood cell (WBC) count at 13,500/μL with neutrophilia (86.3% neutrophils) and a urine test showed four to six WBCs and two to four red blood cells. An abdominal ultrasound performed failed to determine whether the appendix was inflamed or not; there were no pathological findings from the rest of the abdominal examination. An open appendectomy was performed. The macroscopic appearance of his appendix was normal. The pathological examination revealed the lumen to contain *E. vermicularis *without inflammatory infiltrations in the underlying mucosa (Figure 1, Figure 2 and Figure 3). Postoperatively, one oral dose of 100 mg of mebendazole was administered to our patient and his family members and was repeated after 15 days. One year after the operation, our patient was free of symptoms.

## Discussion

Gastrointestinal infection due to *E. vermicularis *occurs worldwide and is considered to be the most common helminth infection [2]. This condition occurs in all ages and socioeconomic levels, but it is most common in children aged five to fourteen years [8]. Yildirim *et al*. [12] reported a mean age of 38 ± 15.75 years in patients who were operated on for acute appendicitis with the pathological examination revealing *E. vermicularis*. Humans are the only natural hosts of pinworms. Embryonated eggs measure 30 μm to 60 μm and are found on fingernails, clothing, house dust and other fomites. After ingestion, the eggs hatch in the stomach and then the coiled larvae appear. Larvae travel to the cecum, where they mature to adult pinworms measuring 1 cm in length. Gravid adult female worms migrate during the night to the perianal region, where they deposit up to 11,000 eggs. Eggs are infective within six hours of ovi deposition. The lifespan of a pinworm is between 11 days and 35 days [8].

Globally, the reported incidence of *E. vermicularis *in patients with symptoms of appendicitis ranges from 0.2% to 41.8% [14]. Unfortunately, there are no studies conducted in Greece, therefore we ignored the incidence of *E. vermicularis *infestation in the Greek population. The role of *E. vermicularis *as a cause of acute appendicitis has been controversial [15]. Some studies confirm the findings of acute or chronic inflammation in appendix specimens found to have pinworms. However, the majority of studies report a lower incidence of inflammatory changes in patients with appendiceal pinworms. This was found to be the case in our study also. A review by Arca *et al*. [8] of the published reports over the last 30 years does not settle this controversy. *E. vermicularis *infestation may cause a clinical picture resembling acute appendicitis by obstructing the lumen or causing a hypersensitivity reaction in the tissue [12]. However, it is not clear whether the invading organism actually causes the inflammation or if the parasites are incidental findings in cases where inflammation is already present [14]. The literature describes that these problems lead to surgery for clinical diagnosed acute appendicitis. The reported rates of inflammation in specimens from appendices infested with *E. vermicularis *range from 13% to 37% [14].

## Conclusions

The finding of *E. vermicularis *in appendectomy histopathological specimens is a rare incident. Parasitic infections rarely cause a clinical image of acute appendicitis, especially in adults. The surgeon must be aware of parasite infection with appendicitis-like symptoms. Careful examination and symptomatology awareness, such as pruritus ani or eosinophilia in the blood examination, and a high level of suspicion might prevent unnecessary appendectomies.

## Consent

Written informed consent was obtained from the patient for publication of this case report and any accompanying images. A copy of the written consent is available for review by the Editor-in-Chief of this journal

## Competing interests

The authors declare that they have no competing interests.

## Authors' contributions

SP and DP performed the main authorship and data collection, VK performed the pathological examination and revised the manuscript. DP, SS and GB reviewed the literature and revised the manuscript. AM assisted in the authorship and revised the manuscript critically. All authors read and approved the final manuscript.
